# Land–ocean nutrient gradients are associated with seasonal shifts in net-collected phytoplankton assemblages in the Yangtze River Estuary exclusive economic zone

**DOI:** 10.3389/fmicb.2026.1897991

**Published:** 2026-08-20

**Authors:** Bangping Deng, Wei Liu, Jun Li, Tao Yang, Jun Shi, Yiming Yuan, Shengming Cheng, Lei Wu, Chuxu Xiong, Lichen Zhao, Xiping Zheng, Lin Zhu

**Affiliations:** 1 East China Sea Ecology Center, and Key Laboratory of Marine Ecological Monitoring and Restoration Technologies, Ministry of Natural Resources, Shanghai, China; 2 China Three Gorges Investment Management Co., Ltd., Shanghai, China; 3 Huaneng (Shanghai) Clean Energy Development Co., Ltd., Shanghai, China; 4 Shanghai Electric Power Co., Ltd., Shanghai, China; 5 Shanghai Electric Wind Power Group Co., Ltd., Shanghai, China; 6 CRCC Harbour & Channel Engineering Bureau Group Co., Ltd., Zhuhai, Guangdong, China

**Keywords:** aquatic microbiology, environmental drivers, exclusive economic zone, seasonal dynamics, Yangtze River Estuary

## Abstract

**Introduction:**

River-influenced continental shelves impose strong seasonal nutrient pressure on phytoplankton assemblages, yet the environmental regulation of phytoplankton assemblages in the outer estuarine zone, and the way their dynamics propagate to higher trophic levels, remains poorly resolved.

**Methods:**

We conducted three cruises in spring, summer, and autumn 2024 at 28 stations in the exclusive economic zone (EEZ) adjacent to the Yangtze River Estuary, characterizing the phytoplankton community together with water quality, surficial sediment geochemistry, and the accompanying zooplankton and macrobenthos as ecosystem context.

**Results:**

The phytoplankton community was strongly seasonal and near-monodominant in summer: the diatom Skeletonema costatum contributed 76.3% of total cell density in spring and 98.4% in summer, then collapsed to 0.7% in autumn, when diversity rose as dominance dispersed among larger diatoms; cell density declined by three orders of magnitude from spring to autumn. These shifts tracked the physicochemical environment. Dissolved inorganic nitrogen exceeded the Class I seawater criterion in 53.6% of spring–summer observations, dissolved inorganic phosphorus exceedance peaked in autumn at 89.3%, and summer bottom-water dissolved oxygen fell to 2.10 mg L−1, consistent with regional hypoxia; trace metals remained below Class I sediment criteria. Zooplankton biomass peaked in summer, whereas macrobenthic standing stock declined 14-fold from summer to autumn, with three stations azoic. Canonical correspondence analysis retained dissolved oxygen and temperature across all biological groups, while nutrient variables were retained only for the planktonic communities.

**Discussion:**

The phytoplankton community thus emerges as the most environmentally responsive component of this shelf ecosystem, underscoring the value of integrated, multi-season monitoring of net-collected microalgal assemblages to support coastal management.

## 1 Introduction

Coastal ecosystems at the interface between large rivers and the open ocean are highly productive but vulnerable to anthropogenic nutrient enrichment. Over recent decades, fertilizer use, wastewater discharge, and land-use change have increased nitrogen and phosphorus inputs to many nearshore seas by several fold (Cloern, 2001; Fang et al., 2025). Elevated nutrient loading can stimulate primary production and alter phytoplankton composition, promote seasonal bottom-water hypoxia, modify sediment biogeochemistry, and degrade benthic habitats (Wang H. et al., 2016; Zhang et al., 2016). Among the biological responders, phytoplankton—the dominant autotrophic microbial community of these waters—are the first to register nutrient change, and their assemblage shifts subsequently propagate to zooplankton and benthic consumers. Because these responses depend on hydrodynamic residence time, water–column stratification, and water–mass interactions, their seasonal dynamics require integrated assessments of water quality, sediments, and biological communities within a common spatiotemporal framework (Cloern, 2001; Liu et al., 2021; Kim et al., 2019; Lheureux et al., 2022).

The Changjiang, or Yangtze River, delivers approximately 9.0 × 10^11^ m^3^ yr.^−1^ of freshwater and large loads of dissolved and particulate materials to the East China Sea (ECS) (Zhang et al., 2007; Duan et al., 2008). Its freshwater plume interacts with the Taiwan Warm Current, Kuroshio subsurface water, and coastal currents, generating a seasonally variable mosaic of water masses across the adjacent shelf (Lian et al., 2016; Liu et al., 2021). Nutrient concentrations in this region have changed substantially: dissolved inorganic nitrogen (DIN) increased from the 1980s to the early 2000s before plateauing, while dissolved inorganic phosphorus (DIP) followed a broadly similar but lagged trajectory, partly affected by phosphorus retention behind the Three Gorges Dam (Ye et al., 2020; Chen et al., 2021; Liu et al., 2022). Persistent and imbalanced N:P loading has made the Changjiang estuary and adjacent waters a major hotspot of seasonal hypoxia, with summer bottom-water dissolved oxygen often falling below 3 mg L^−1^ over more than 10,000 km^2^ (Li et al., 2002; Wang H. et al., 2016; Wang et al., 2017).

Biological responses are equally seasonal, and the phytoplankton community is a dynamic microalgal component of this system. The diatom *Skeletonema costatum* frequently dominates spring and summer phytoplankton assemblages, and its exported bloom biomass contributes to bottom-water oxygen consumption (Xu et al., 2019; Sun et al., 2022; He et al., 2025). The composition and diversity of these microalgal assemblages can therefore shift markedly within a single season in response to nutrient and oxygen gradients. Zooplankton assemblages, which graze on this primary production, shift with temperature and salinity gradients (Wang L. et al., 2016; Li et al., 2026), whereas macrobenthos integrate cumulative environmental stress, with dissolved oxygen acting as a key threshold variable (Huang et al., 2022; Han et al., 2025). Sediment geochemistry is shaped by terrestrial inputs, grain-size sorting, and diagenetic recycling, producing spatial gradients in organic matter and trace metals from the estuary to the outer shelf (Cao et al., 2015; Yang et al., 2015; Xu et al., 2018).

Despite extensive research, the environmental regulation of the phytoplankton community in the outer estuarine zone, and the way its seasonal dynamics relate to the accompanying zooplankton and benthic communities, remains insufficiently resolved. Phytoplankton, zooplankton, and macrobenthos, together with their physicochemical setting, have rarely been assessed simultaneously across seasons within a unified design, and spatial coverage has been concentrated in the inner estuary and nearshore shelf, with fewer surveys extending into the EEZ, where the land–ocean gradient weakens but persists. We hypothesized that, across this gradient, the phytoplankton community would be the most environmentally responsive component of the ecosystem, that dissolved oxygen and temperature would act as drivers shared across trophic levels, and that dissolved nutrients would structure the planktonic communities more strongly than the benthos. Testing this requires resolving seasonal community structure and its environmental correlates within a common spatiotemporal framework.

To address this gap, we conducted three seasonal cruises in 2024 across 28 stations in the EEZ adjacent to the Yangtze River Estuary, resolving the phytoplankton community alongside its physicochemical setting and the accompanying zooplankton and macrobenthos. The objectives were to: (1) characterize the seasonal structure and diversity of the phytoplankton community and place the accompanying zooplankton and benthic communities in this context; (2) characterize the seasonal water-column and sediment environment and evaluate compliance with Chinese national quality standards; and (3) identify community–environment associations across trophic levels. We first describe the seasonal water and sediment environment, then the biological communities, and finally relate community structure to environmental gradients by constrained ordination, so as to clarify how land–ocean gradients are associated with the seasonal trajectory of the phytoplankton community and the wider shelf ecosystem.

## 2 Materials and methods

### 2.1 Survey design and sampling

**Figure 1:**
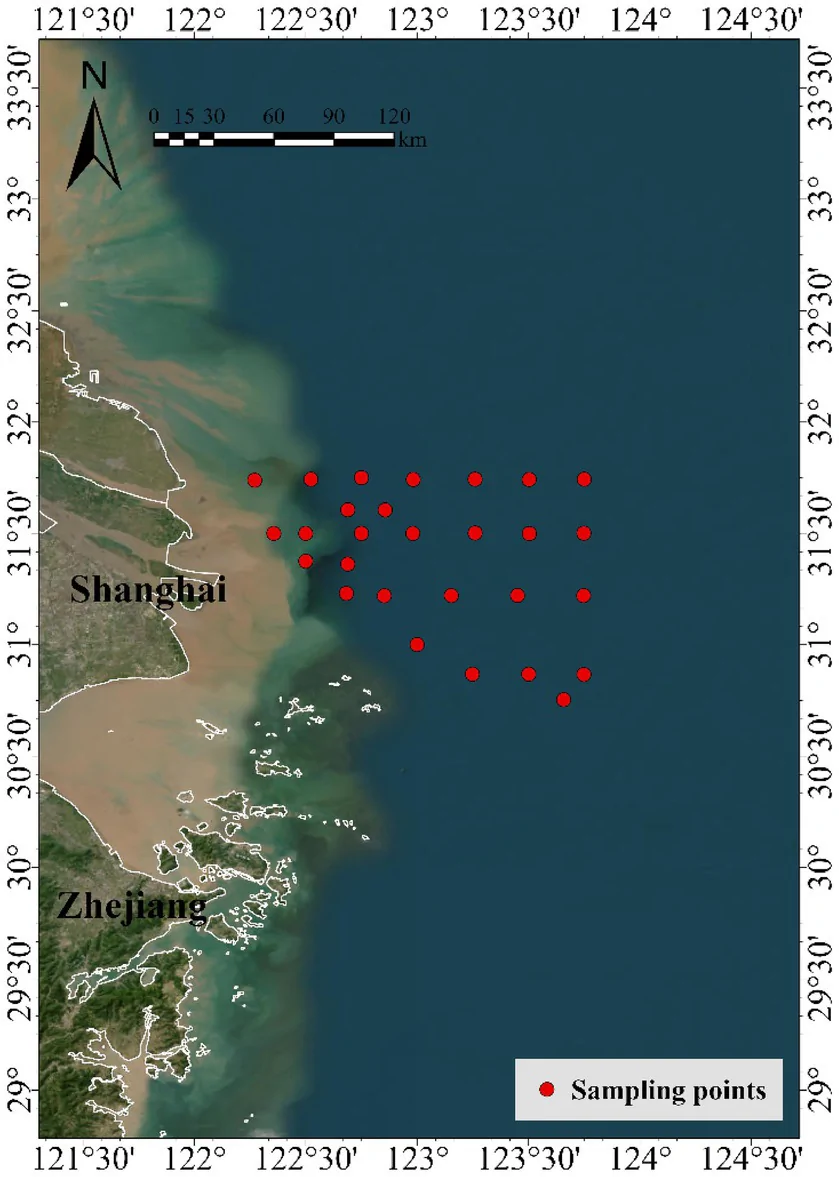
Location of the 28 sampling stations in the exclusive economic zone (EEZ) adjacent to the Yangtze River Estuary, East China Sea. The station array spans approximately 122.27°–123.75° E and 30.75°–31.75° N, covering water depths from approximately 14 to 60 m. The satellite base map illustrates the turbid Yangtze freshwater plume extending seaward from the estuary. All 28 stations were sampled during the summer (July–August 2024) and autumn (October–November 2024) cruises; during the spring cruise (April 2024), full-element biological sampling was conducted at 17 of the 28 stations.

Three cruises were conducted in 2024 across the EEZ adjacent to the Yangtze River Estuary: 21–25 April, 15 July–6 August, and 9 October–11 November. Each cruise was completed as a continuous campaign to reduce within-season temporal heterogeneity. The survey domain extended from approximately 122.27° to 123.75° E and 30.75° to 31.75° N, covering depths of 14–60 m. The area includes the main pathway of the Yangtze freshwater plume, the residual flow of the Taiwan Warm Current, and the seaward edge of the coastal mixing zone (Lian et al., 2016; Liu et al., 2021). Twenty-eight stations were arranged in a quasi-uniform grid to resolve cross-shelf and meridional gradients (Figure 1).

In spring, full-element sampling—water quality, sediment, phytoplankton, zooplankton, and macrobenthos—was conducted at 17 stations; the remaining 11 stations were sampled only for water and sediment. In summer and autumn, full-element sampling was conducted at all 28 stations. Seasonal biological comparisons were based on station-level metrics from the available full-element stations, with no imputation for the 11 spring stations without biological samples. Constrained ordinations used only samples with matched biological and environmental data, and the unequal spring coverage is treated as a limitation when interpreting seasonal biological contrasts.

At each station, water was collected from the surface layer at 0.5 m, the 10 m layer, and the near-bottom layer, approximately 1–2 m above the seabed, using Niskin bottles mounted on a CTD rosette. At stations shallower than 15 m, only surface and bottom samples were collected. Sediments were retrieved using a 0.1 m2 box corer, and the upper 0–2 cm layer was subsampled. Samples were processed within 30 min on board; water and biological samples were stored at 4 °C, and sediment subsamples at −20 °C.

### 2.2 Sample collection and laboratory analyses

Twenty-two water quality variables were measured: temperature, salinity, pH, dissolved oxygen (DO), suspended particulate matter (SPM), chlorophyll-a, total organic carbon (TOC), permanganate index (COD_Mn_), ammonium, nitrate, nitrite, DIN, DIP, petroleum hydrocarbons, and Cu, Pb, Zn, Cd, Hg, As, and Cr. Temperature, salinity, pH, and DO were measured *in situ* using a Sea-Bird SBE 25 CTD. Other variables were analyzed following GB 17378.4–2007 and GB/T 12763.4–2007. Quality control included field blanks, transport blanks, and duplicate samples at approximately 10% frequency. Recoveries were 85%–115% for trace metals and 80%–120% for nutrients.

Nineteen sediment variables were analyzed following GB 17378.5–2007: grain-size composition, median grain size, TOC, total nitrogen (TN), total phosphorus (TP), sulfides, petroleum hydrocarbons, Cu, Pb, Zn, Cd, Hg, As, Cr, hexachlorocyclohexane (HCH), and dichlorodiphenyltrichloroethane (DDT).

Phytoplankton were collected by vertical hauls from bottom to surface using a 77 μm plankton net, fixed in 1% Lugol’s solution, and identified to the lowest practicable taxonomic level using the Utermöhl method (Utermöhl, 1958). Cell densities were standardized to cells m^−3^ using the filtered water volume for each vertical haul, and values should be interpreted as densities of the retained net-collected fraction rather than whole-water phytoplankton concentrations. This design characterizes larger and colonial phytoplankton, including dominant diatoms, but does not quantitatively resolve picophytoplankton, small flagellates, bacteria, archaea, or many non-pigmented protists. Zooplankton were collected using a 505 μm plankton net with a 0.5 m2 mouth area, preserved in 5% buffered formaldehyde, and counted under a stereomicroscope. Macrobenthos were collected from three replicate grabs per station, sieved through 0.5 mm mesh, fixed in 7% buffered formaldehyde, stained with Rose Bengal, and identified to species level when possible.

The Shannon–Wiener diversity index was calculated as:

$$H^{\prime }=-\sum_{i=1}^{S}P_{i}\ln P_{i}$$

Where *S* is the total number of species at a given station, $P_{i}$ is the proportional abundance of species *i*, and *H′* is the Shannon–Wiener diversity index (calculated using the natural logarithm).

### 2.3 Water and sediment quality assessment

Seawater and surficial sediment quality was assessed using the single-factor pollution index (GB 3097–1997; GB 18668–2002):

$$P_{i}=\frac{C_{i}}{S_{i}}$$

Where *P_i_* is the pollution index of variable *i*, *C_i_* is the measured concentration of variable *i* at a given station and depth, and *S_i_* is the corresponding evaluation criterion. For DO, the pollution index was computed as:

$$P_{\mathrm{DO}}=\frac{|DO_{f}-\mathrm{DO}|}{DO_{f}-S_{\mathrm{DO}}}$$

$$P_{\mathrm{DO}}=10-9\times \frac{\mathrm{DO}}{S_{\mathrm{DO}}}$$

Where *DO_f_* is the saturated DO concentration at the *in situ* temperature and salinity, calculated following GB 17378.4–2007. A value of *P_i_* ≤ 1 indicates compliance with the criterion, while *P_i_* > 1 indicates exceedance. Seawater quality was classified against the Class I criteria of GB 3097–1997; sediment quality against the Class I criteria of GB 18668–2002.

### 2.4 Statistical analyses

Analyses were performed in R 4.5.1 (R Core Team, 2025). Normality and homogeneity of variance were tested using Shapiro–Wilk and Levene’s tests. Because most seasonal variables were non-normal, heteroscedastic, or based on unequal seasonal sample sizes, the final seasonal comparisons reported in the main text used Kruskal–Wallis tests, followed by Dunn tests with Bonferroni correction for water and sediment variables, or pairwise Wilcoxon tests with Benjamini–Hochberg correction for biological metrics. Statistical significance was set at *α* = 0.05; trends with 0.05 ≤ *p* < 0.10 are reported as marginal.

Community–environment relationships were analyzed using constrained ordination in the vegan package (Oksanen et al., 2007). Species-by-station abundance matrices were constructed for each biological group across seasons; species occurring at fewer than 5% of stations were excluded. Abundance data were Hellinger-transformed to reduce the leverage of highly abundant taxa and to make the ordination less dominated by total-count differences among stations (Legendre and Gallagher, 2001). DCA was used as a diagnostic of compositional gradient length; because the first-axis gradient exceeded 3 SD units for all three biological groups, CCA was used for the final constrained ordinations. The CCA results are interpreted as community–environment associations rather than as direct tests of causal control.

For phytoplankton and zooplankton, the environmental matrix included six surface-water variables: temperature, DO, DIN, DIP, COD_Mn_, and SPM. For macrobenthos, the same six near-bottom water variables were supplemented with seven sediment variables available across seasons: OC, sulfide, Cu, Zn, Pb, Cr, and As. Salinity, sediment TN, TP, and grain-size fractions were excluded because of missing seasonal data. Collinearity was screened using variance inflation factors, with VIF > 10 as the exclusion threshold. Significance was tested using 999 Monte Carlo permutations, and forward selection identified the parsimonious variable set using ordistep.

Comparisons with published baselines were descriptive because historical surveys differed in sampling design, gear, and analytical protocols.

## 3 Results

### 3.1 Seasonal patterns of water quality

Surface temperature increased from 15.31 °C in spring to 28.22 °C in summer, then declined to 21.67 °C in autumn. Bottom-layer means were 12.91, 21.59, and 22.10 °C. Domain-averaged salinity was 29.58, 29.37, and 32.68 across the three seasons, with summer surface salinity reaching a minimum of 3.84 at inner-shelf stations under peak Yangtze discharge.

**Figure 2:**
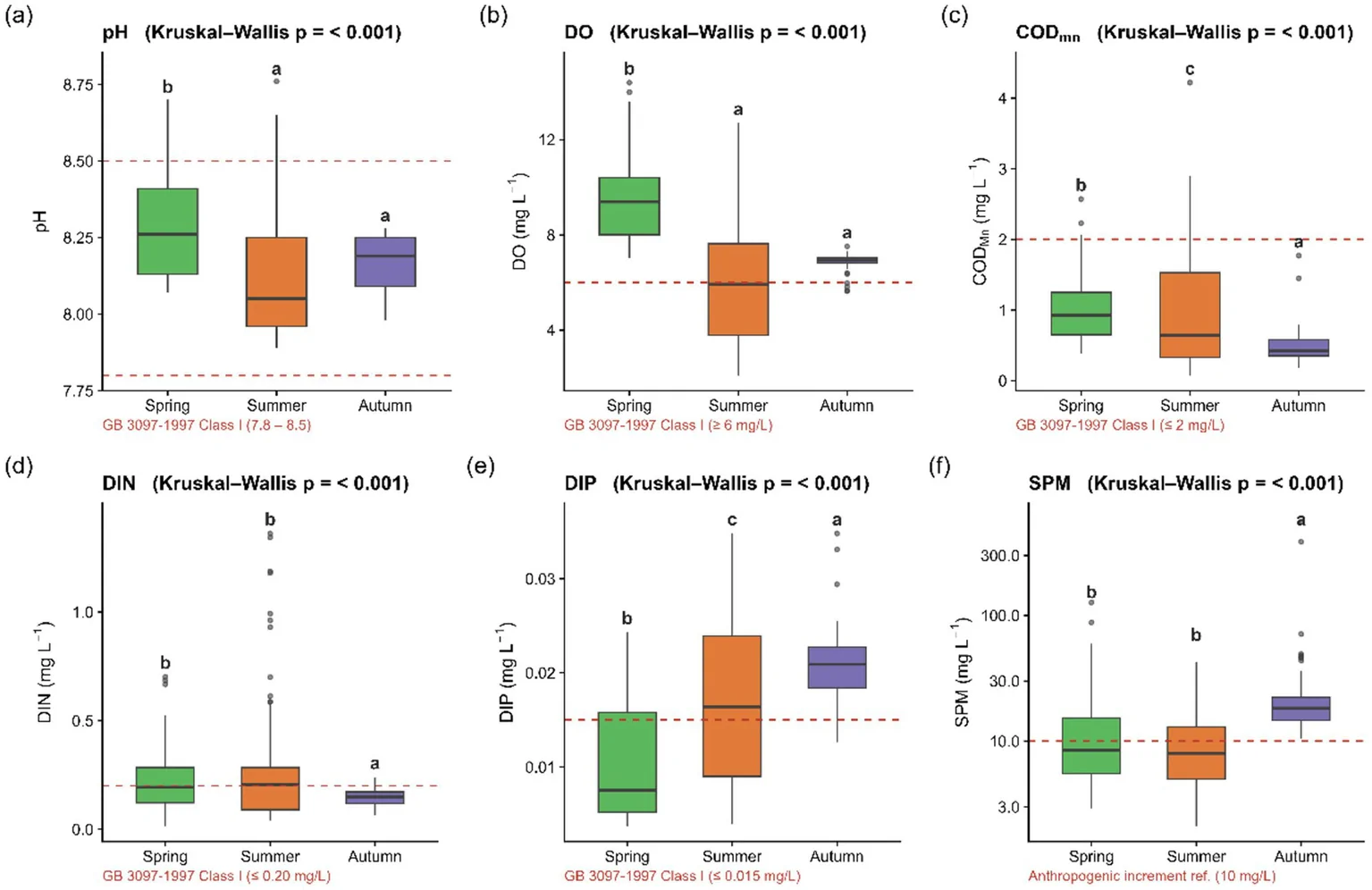
Seasonal variation in six core water quality parameters across the Yangtze River Estuary EEZ during 2024. Panels show **(a)** pH, **(b)** dissolved oxygen (DO, mg L^−1^), **(c)** permanganate index (COD_Mn_, mg L^−1^), **(d)** dissolved inorganic nitrogen (DIN, mg L^−1^), **(e)** dissolved inorganic phosphorus (DIP, mg L^−1^), and **(f)** suspended particulate matter (SPM, mg L^−1^). Data from all station–depth observations are pooled within each season. Boxes represent the interquartile range with the median line; individual observations are shown as jittered points. Dashed horizontal lines indicate the Class I criteria of GB 3097–1997 (or the anthropogenic increment reference of 10 mg L^−1^ for SPM). Compact letter displays denote significant pairwise differences (Kruskal–Wallis test followed by pairwise Wilcoxon rank-sum tests with Benjamini–Hochberg correction; groups sharing the same letter do not differ significantly at *p* < 0.05).

All six core water quality parameters differed significantly among seasons (Kruskal–Wallis, all *p* < 0.001; Figure 2). pH was higher in spring (8.29) than in summer (8.13) and autumn (8.17), which did not differ significantly. DO showed a similar grouping: spring values averaged 9.51 mg L^−1^, exceeding summer and autumn means of 5.85 and 6.89 mg L^−1^. The seasonal mean, however, obscured strong summer stratification. Bottom-layer DO averaged 3.45 mg L^−1^ and reached 2.10 mg L^−1^, close to the conventional hypoxic threshold of 2 mg L^−1^; by autumn, bottom-layer DO exceeded 5.64 mg L^−1^.

COD_Mn_ declined from spring to autumn, with means of 1.03, 0.92, and 0.48 mg L^−1^, all below the Class I criterion of 2.0 mg L^−1^. SPM was highest in autumn, averaging 31.7 mg L^−1^ and reaching 560 mg L^−1^, compared with 14.6 mg L^−1^ in spring and 9.8 mg L^−1^ in summer.

DIN and DIP showed contrasting seasonal trajectories. DIN remained high in spring and summer, with means of 0.232 and 0.283 mg L^−1^, before declining to 0.143 mg L^−1^ in autumn. Across spring and summer, DIN exceeded the Class I threshold of 0.20 mg L^−1^ in 53.6% of station-depth observations, mainly at inner-shelf stations. DIP increased from 0.011 mg L^−1^ in spring to 0.017 mg L^−1^ in summer and 0.021 mg L^−1^ in autumn; 89.3% of autumn stations exceeded the Class I threshold of 0.015 mg L^−1^.

Trace metals and petroleum hydrocarbons remained below Class I criteria across seasons. Only Cu and As showed marginally higher spring values. Chlorophyll-a peaked in spring and summer, with a spring mean of 4.16 μg L^−1^ and a summer surface maximum of 32.8 μg L^−1^, before declining to 0.44 μg L^−1^ in autumn.

### 3.2 Seasonal patterns of sediment geochemistry

**Figure 3:**
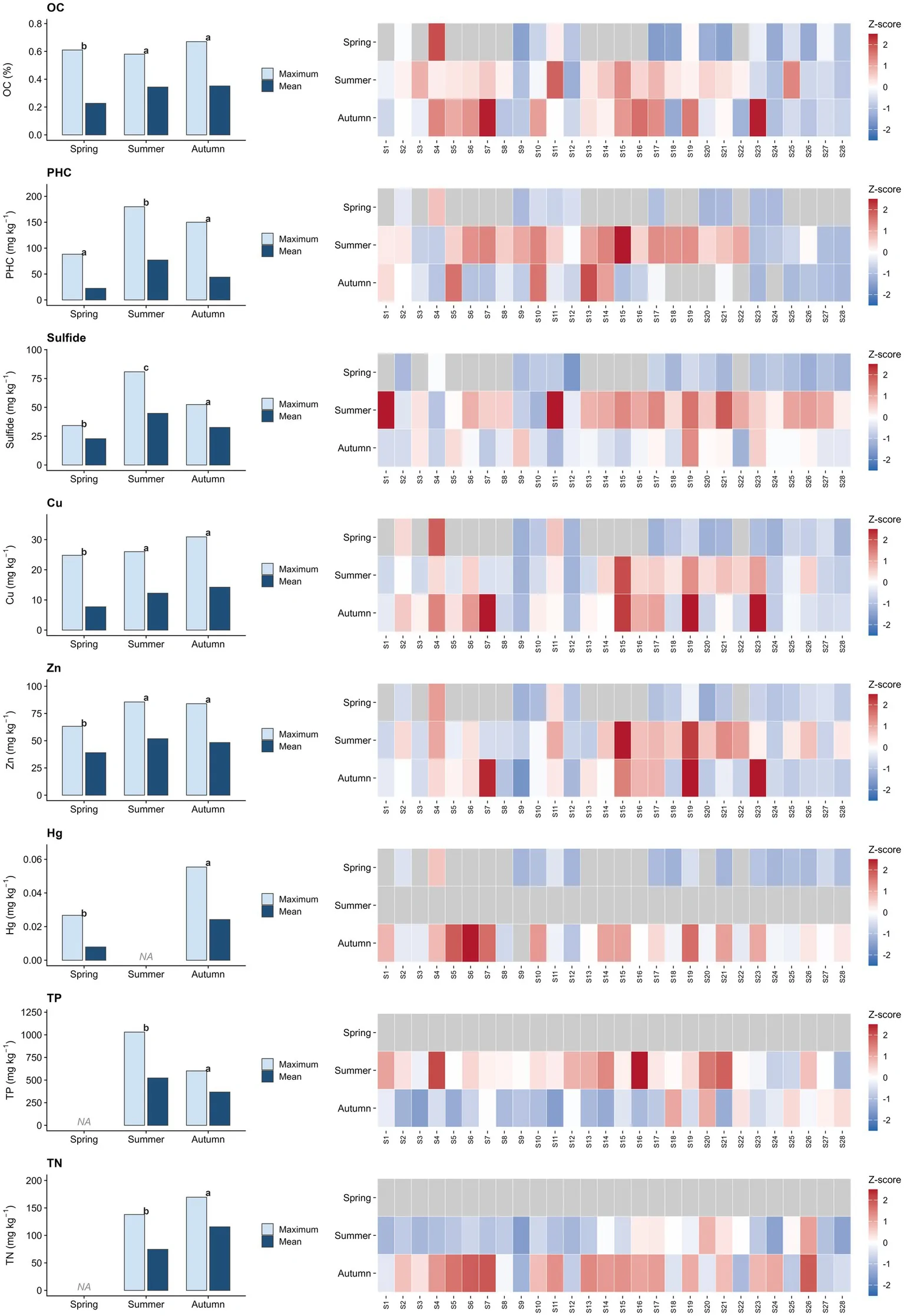
Seasonal comparison of eight principal sediment quality parameters in the Yangtze River Estuary EEZ during 2024: Organic carbon (OC), petroleum hydrocarbons (PHC), sulfide, copper (Cu), zinc (Zn), mercury (Hg), total phosphorus (TP), and total nitrogen (TN). For each parameter, the left panel displays the seasonal maximum (light bar) and mean (dark bar) concentrations; compact letter displays denote significant pairwise differences (Kruskal–Wallis test followed by Dunn’s test with Bonferroni correction; *p* < 0.05). “NA” indicates that the season was excluded from the statistical comparison owing to missing data (Hg lacked summer data; TP and TN lacked spring data). The right panel shows station-level *Z*-score heatmaps standardized within each parameter across all valid observations; grey cells indicate missing data. Stations (S1–S28) are ordered by increasing distance from the Yangtze River mouth (31.40° N, 122.10° E) to highlight the land-to-ocean gradient.

The 19 surficial sediment variables showed three seasonal patterns (Figure 3). Data coverage varied: Hg lacked summer data, TP and TN lacked spring data, and PHC had reduced coverage in spring and autumn.

The first group peaked in summer. PHC averaged 76.9 μg g^−1^ in summer, exceeding autumn and spring values of 43.9 and 22.3 μg g^−1^. Sulfide increased from 22.8 mg kg^−1^ in spring to 32.6 mg kg^−1^ in autumn and 44.8 mg kg^−1^ in summer. TP, measured only in summer and autumn, was higher in summer than autumn, with means of 522.8 and 367.2 mg kg^−1^. These summer maxima coincided with peak Yangtze discharge.

The second group showed elevated summer and autumn values relative to spring. OC averaged 0.344 mg g^−1^ in summer and 0.351 mg g^−1^ in autumn, both exceeding the spring mean of 0.227 mg g^−1^. Cu and Zn followed the same pattern: Cu averaged 7.7, 12.2, and 14.2 μg g^−1^, and Zn averaged 39.0, 51.8, and 48.3 μg g^−1^ in spring, summer, and autumn, respectively.

The third group showed higher autumn values based on available data. TN was higher in autumn than summer, with means of 115.6 and 74.6 mg kg^−1^. Hg was higher in autumn than spring, with means of 0.024 and 0.008 mg kg^−1^. Missing summer Hg and spring TN data prevented complete seasonal reconstruction.

Pb, Cd, As, and Cr remained within narrow ranges and below Class I criteria. HCH and DDT were below Class I thresholds at all stations, although detection frequencies were higher in summer, suggesting low-level input or resuspension of historical residues. Spatially, nearshore stations, especially S1–S9, had higher *Z*-scores for OC, PHC, and Cu, whereas offshore stations showed lower and more uniform concentrations, consistent with a land-to-ocean enrichment gradient.

### 3.3 Seasonal patterns of biological communities

The phytoplankton community, together with the accompanying zooplankton and macrobenthos, showed broadly coherent seasonal trajectories: summer generally supported higher standing stock, while autumn marked a widespread decline (Table 1). The net-collected phytoplankton assemblage is treated here as the primary biological focus, with zooplankton and macrobenthos providing higher-trophic-level context; diversity responses differed among groups.

**Table 1:** Seasonal variation in biological community structure across the Yangtze River Estuary EEZ during 2024.

Community	Parameter	Spring	Summer	Autumn	*H*
Phytoplankton	*n*	17	28	28	—
	*S*	79	110	86	—
	Density (cells m^−3^)	7.32 × 10^7a^	5.34 × 10^7b^	4.00 × 10^4c^	49.42***
	*H*′	1.10 ± 0.40^a^	0.82 ± 0.62^a^	1.87 ± 0.55^b^	31.56***
	Dominant species	*S. costatum* (76.3%)	*S. costatum* (98.4%)	*C. wailesii* (30.4%)	—
Zooplankton	*n*	17	23	28	—
	*S*	26	79	33	—
	Density (ind m^−3^)	465.2^a^	414.5^a^	40.4^b^	46.42***
	Biomass (mg m^−3^)	293.9^b^	642.1^a^	205.4^c^	36.11***
	*H*′	0.92 ± 0.32^c^	2.29 ± 0.49^a^	1.96 ± 0.47^b^	37.80***
	Dominant species	*C. sinicus* (93.8%)	*C. sinicus* (22.2%)	*E. concinna* (20.5%)	—
Macrobenthos	*n*	17	28	28	—
	*S*	75	138	33	—
	Density (ind m^−2^)	249.4^b^	533.1^a^	37.1^c^	41.81***
	Biomass (g m^−2^)	6.23^b^	25.55^a^	1.81^c^	36.19***
	*H*′	2.18 ± 0.64^a^	2.56 ± 0.79^a^	1.07 ± 0.71^b^	30.98***
	Dominant species	*H. filiformis* (16.7%)	*S. asiatica* (12.6%)	*H. filiformis* (27.6%)	—

Values with different superscript letters within a row differ significantly (*p* < 0.05, pairwise Wilcoxon test with Benjamini–Hochberg correction). *S*, total species count; *H*′, Shannon–Wiener diversity index (mean ± SD, natural logarithm); *H*, Kruskal–Wallis test statistic. ***p < 0.001. Dominant species percentages are proportions of domain-total density excluding larval forms.

**Figure 4:**
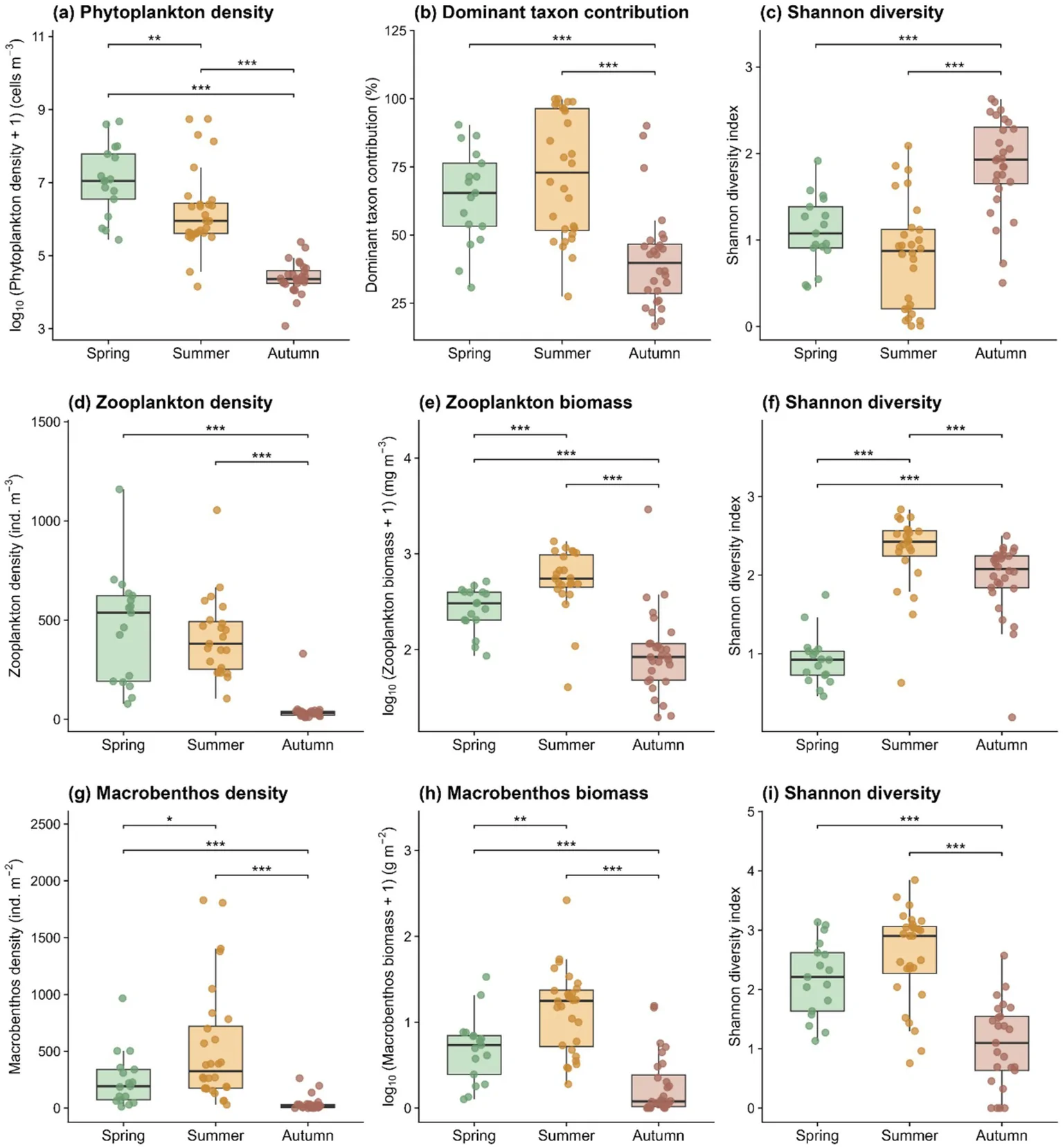
Seasonal variation in community-level metrics for three biological groups in the Yangtze River Estuary EEZ during 2024. The 3 × 3 matrix is organized by community group (rows) and metric (columns): Phytoplankton **(a–c)**, zooplankton **(d–f)**, and macrobenthos **(g–i)**. Columns show density **(a, d, g)**, dominant taxon contribution or biomass **(b, e, h)**, and Shannon–Wiener diversity index *H′* **(c, f, i)**. Phytoplankton density and macrobenthic biomass are displayed on a log₁₀(*x* + 1) scale; zooplankton biomass is likewise log_10_-transformed. Boxes represent the interquartile range with the median line; individual station values are shown as jittered points. Significance brackets indicate pairwise differences from Kruskal–Wallis tests followed by pairwise Wilcoxon rank-sum tests with Benjamini–Hochberg correction (*p < 0.05, **p < 0.01, ***p < 0.001). Spring *n* = 17, summer *n* = 28 (zooplankton *n* = 23), autumn *n* = 28.

A total of 79, 110, and 86 phytoplankton species were recorded in spring, summer, and autumn. Cell density declined from mean 7.32 × 10^7^ cells m^−3^ in spring to 5.34 × 10^7^ cells m^−3^ in summer and 4.00 × 10^4^ cells m^−3^ in autumn, with all seasons significantly different (Figure 4a). *S. costatum* dominated spring and summer, contributing 76.3% and 98.4% of total cell density, respectively. In autumn, its contribution fell to 0.7%, and dominance shifted to *Coscinodiscus wailesii* (30.4%), *Thalassiothrix frauenfeldii* (24.5%), and *Biddulphia sinensis* (17.4%) (Figure 4b). Phytoplankton (H′) was highest in autumn (1.87), compared with spring (1.10) and summer (0.82), reflecting the collapse of *S. costatum* dominance (Figure 4c).

Zooplankton richness was 26, 79, and 33 species in spring, summer, and autumn. Numerical density was similar in spring and summer, averaging 465.2 and 414.5 ind m^−3^, but declined to 40.4 ind m^−3^ in autumn (Figure 4d). Biomass peaked in summer at 642.1 mg m^−3^, exceeding spring and autumn values of 293.9 and 205.4 mg m^−3^, indicating a shift toward larger-bodied taxa (Figure 4e). The spring community was dominated by *Calanus sinicus* at 93.8% of total density, whereas summer dominance was distributed among 11 species. Zooplankton (H′) was highest in summer (2.29), followed by autumn (1.96) and spring (0.92) (Figure 4f).

Macrobenthic richness was 75, 138, and 33 species in spring, summer, and autumn. Density and biomass peaked in summer and declined sharply in autumn. Density averaged 249.4, 533.1, and 37.1 ind m^−2^, and biomass averaged 6.23, 25.55, and 1.81 g m^−2^ across spring, summer, and autumn (Figures 4g, h). The summer-to-autumn density ratio of 14:1 was the strongest seasonal transition observed. Three autumn stations recorded no macrobenthic individuals. Macrobenthic (*H*′) was relatively high in spring and summer, at 2.18 and 2.56, but declined to 1.07 in autumn (Figure 4i).

Across groups, autumn consistently represented the minimum in standing stock. Diversity, however, responded differently: phytoplankton (*H*′) increased after the collapse of a single dominant species, whereas zooplankton and macrobenthic diversity declined with standing stock.

### 3.4 Community–environment relationships

DCA first-axis gradient lengths were 3.75, 3.18, and 7.04 SD for phytoplankton, zooplankton, and macrobenthos; CCA was therefore used for all groups (Table 2). After VIF screening, all candidate environmental variables were retained for forward selection.

**Table 2:** Summary of canonical correspondence analysis (CCA) results for three biological community groups.

Parameter	Phytoplankton	Zooplankton	Macrobenthos
No. of samples	68	63	68
No. of species (after filtering)	83	74	91
DCA gradient length (SD)	3.75	3.18	7.04
Ordination method	CCA	CCA	CCA
No. of candidate variables	6	6	13
Variables retained	DO, Temp, DIN, DIP, COD_Mn_	Temp, COD_Mn_, DIN, DIP, DO	DO, Temp, Sed-As
Total constrained inertia (%)	33.0	39.8	9.2
CCA1 explained (%)	18.1	19.9	3.6
CCA2 explained (%)	7.3	11.4	3.5
Model pseudo-*F*	6.11	7.52	2.16
Model *p*	0.001	0.001	0.001

Species occurring at fewer than 5% of stations were excluded prior to analysis. Abundance data were Hellinger-transformed. Forward selection was performed using the *ordistep* function with 999 Monte Carlo permutations. Candidate environmental variables comprised six surface-layer water quality variables for phytoplankton and zooplankton, and six bottom-layer water quality variables plus seven surficial sediment variables for macrobenthos. Salinity was excluded owing to unavailability of spring data.

**Figure 5:**
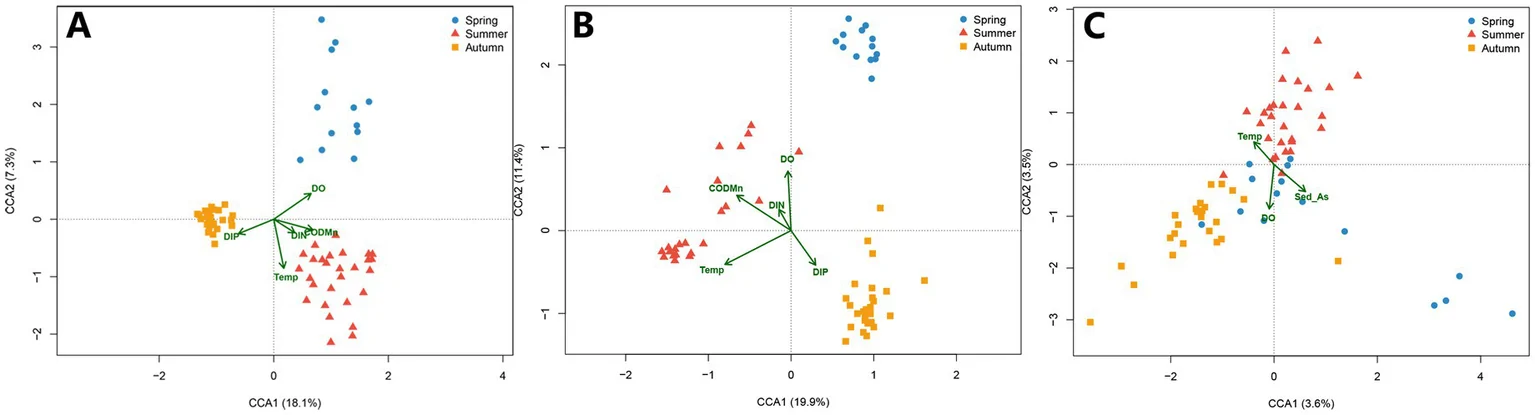
Canonical correspondence analysis (CCA) biplots for **(A)** phytoplankton, **(B)** zooplankton, and **(C)** macrobenthos in the Yangtze River Estuary EEZ. Station scores are coded by season: spring (blue circles), summer (red triangles), and autumn (orange squares). Green arrows represent environmental variables retained by forward selection (*p* < 0.05, 999 Monte Carlo permutations): Temp, temperature; DO, dissolved oxygen; DIN, dissolved inorganic nitrogen; DIP, dissolved inorganic phosphorus; COD_Mn_, permanganate index; Sed_As_, sediment arsenic. Arrow length and direction indicate the strength and direction of the variable’s correlation with the ordination axes. The percentage of total inertia explained by each axis is shown in parentheses. Abundance data were Hellinger-transformed prior to analysis; species occurring at fewer than 5% of stations were excluded.

For phytoplankton, CCA included 68 samples and 83 species after filtering. Five variables—DO, temperature, DIN, DIP, and COD_Mn_—explained 33.0% of community variation, with CCA1 and CCA2 accounting for 18.1 and 7.3% (*F* = 6.11, *p* = 0.001; Figure 5A). Seasonal groups separated along CCA1, with summer stations associated with temperature and DIN and spring stations with DO. The near-orthogonal DIN and DO vectors suggest that nutrient enrichment and oxygen depletion represented complementary gradients.

For zooplankton, CCA included 63 samples and 74 taxa after filtering. Five variables—temperature, COD_Mn_, DIN, DIP, and DO—explained 39.8% of variation, the highest among the three groups. CCA1 and CCA2 explained 19.9 and 11.4% (*F* = 7.52, *p* = 0.001). Seasonal separation resembled the phytoplankton pattern, although summer samples showed greater within-season scatter. The DIP vector was oriented toward autumn stations, consistent with the seasonal DIP increase (Figure 5B).

For macrobenthos, CCA included 68 samples and 91 taxa after filtering, and the environmental matrix included six water and seven sediment variables. Forward selection retained DO, temperature, and sediment As, explaining 9.2% of variation. CCA1 and CCA2 accounted for 3.6 and 3.5% (*F* = 2.16, *p* = 0.001). The lower explained variance was consistent with the long DCA gradient length, indicating that the retained variables summarized only a small part of benthic community variation, with most variation likely related to unmeasured drivers such as larval supply, biotic interactions, and fine-scale substrate heterogeneity (Figure 5C).

DO and temperature were the only variables retained across all three biological groups, suggesting that they were common axes of seasonal community–environment association. DIN and DIP were retained for both planktonic groups but not for macrobenthos, suggesting that dissolved nutrients were more directly associated with planktonic communities. SPM was not selected for any group.

## 4 Discussion

### 4.1 Seasonal nutrient dynamics and hypoxia

The contrasting seasonal trajectories of DIN and DIP reflect the combined influence of riverine nutrient delivery, water-mass mixing, and biogeochemical cycling (Wei et al., 2020). DIN remained high in spring and summer, consistent with riverine loading during the high-discharge period, whereas DIP increased steadily toward autumn. This divergence agrees with long-term regional trends showing that DIN is largely controlled by river discharge, while DIP is additionally affected by phosphorus retention behind the Three Gorges Dam and sedimentary recycling (Ye et al., 2020; Liu et al., 2022). Sediment nitrogen cycling also provides relevant process context for this seasonal nutrient pattern: denitrification and anammox can remove reactive nitrogen from estuarine-shelf sediments, whereas DNRA and organic nitrogen mineralization can retain nitrogen in ammonium form and potentially sustain internal nutrient availability (Lin et al., 2017a, 2017b; Yao et al., 2026). The autumn increase in DIP likely reflects benthic phosphorus release from organically enriched sediments and reduced influence of the low-phosphorus Changjiang plume as discharge weakens (Liu et al., 2020).

The seasonally offset exceedance windows have direct management implications. DIN exceeded the Class I threshold in 53.6% of spring–summer observations, whereas DIP exceedance reached 89.3% in autumn. A single-season survey would therefore underestimate nutrient pressure in the EEZ. The observed N:P imbalance—nitrogen enrichment during the discharge season and phosphorus enrichment in autumn—resembles nutrient shifts reported for other Chinese semi-enclosed seas, where elevated N:P ratios have been linked to seasonal hypoxia (Qu et al., 2025).

Summer bottom-water DO patterns were consistent with hypoxia off the Changjiang estuary (Li et al., 2002; Wang H. et al., 2016; Wang et al., 2017; Luo et al., 2018; Miao et al., 2023). The minimum DO of 2.10 mg L^−1^ places the EEZ stations near the seaward margin of the hypoxic zone, indicating that oxygen depletion extends beyond the inner shelf during summer. The co-occurrence of high DIN, near monodominance of *S. costatum*, and bottom-water oxygen depletion is consistent with, but does not by itself demonstrate, the eutrophication-driven hypoxia framework (Cloern, 2001; Wang et al., 2017). By autumn, bottom-layer DO had recovered above 5.64 mg L^−1^, coinciding with the collapse of *S. costatum*, which is compatible with reduced bloom-derived organic matter supply and enhanced mixing; direct process-rate measurements would be required to quantify their relative contributions.

Trace metals and petroleum hydrocarbons remained below Class I criteria, consistent with recent evidence of reduced riverine metal inputs after implementation of the Yangtze River Protection Law (Wang et al., 2023). Nutrient enrichment, rather than toxic contamination, therefore appears to be the main water-quality concern in the Changjiang EEZ.

### 4.2 Sediment geochemistry and terrestrial signals

Sediment geochemistry showed seasonal patterns linked to material delivery, retention, and diagenesis. The summer maxima of PHC, sulfide, and TP coincided with peak Yangtze discharge and likely reflected enhanced delivery of particulate organic matter. Elevated sulfide under summer low-oxygen conditions is consistent with intensified sulfate reduction (Yao et al., 2014), while higher TP may reflect riverine particulate phosphorus input and release of iron-bound phosphorus under reducing conditions (Liu et al., 2020). In nitrogen-rich estuarine and shelf sediments, organic matter, nitrate availability, ammonium, sulfide, and Fe(II) can jointly regulate denitrification, anammox, DNRA, nitrification, and ammonium immobilization (Lin et al., 2017a, 2017b; Wei et al., 2020); these linked processes provide a plausible pathway through which flood-season deposition may influence later nutrient availability.

OC, Cu, and Zn remained elevated from summer to autumn, suggesting that flood-season inputs and phytoplankton-derived organic matter persisted on the seabed. This pattern is consistent with the retention of organic matter and trace metals by fine-grained sediments (Cao et al., 2015; Xu et al., 2018; Li, 2025). The persistence of elevated values indicates that flood-season depositional signals were not rapidly removed during the autumn transition.

Higher autumn TN and Hg values point to lagged diagenetic processes, although data gaps limit interpretation. TN enrichment may reflect mineralization of organic nitrogen exported during spring and summer blooms (Yang et al., 2015). The Hg pattern is consistent with mercury binding to organic matter and sulfide phases (Ren et al., 2026), but the absence of summer Hg data prevents a full seasonal assessment. Despite these organic and nutrient-related changes, Pb, Cd, As, and Cr remained below Class I criteria, and organochlorine pesticides were also low. Thus, sediment variability in the Changjiang EEZ was driven mainly by organic and nutrient-related processes rather than toxic-metal enrichment.

### 4.3 Biological reorganization and diversity divergence

The phytoplankton community and the accompanying zooplankton and macrobenthos shared a broad seasonal pattern of high summer standing stock and autumn decline, but their dominance structures and diversity responses differed. This indicates that the seasonal reorganization of the microalgal community, and its propagation to higher trophic levels, was mediated by trophic-level-specific processes rather than a single environmental pathway.

The summer near monodominance of *S. costatum*, which contributed 98.4% of total phytoplankton cell density, represents a strong simplification of the net-collected phytoplankton assemblage. Previous surveys have identified *S. costatum* as a dominant spring–summer species, usually contributing 40%–80% of abundance (Xu et al., 2019; Sun et al., 2022). The higher dominance in 2024 is consistent with elevated DIN, warm surface waters, and stratification favoring its rapid growth over competing taxa. Because exported *S. costatum* biomass contributes to bottom-water oxygen consumption (Wang et al., 2017), its concurrence with the DO minimum is consistent with a bloom-hypoxia linkage, although direct production and respiration measurements would be needed to establish the process quantitatively. In autumn, the decline of *S. costatum* and increased contributions from *C. wailesii*, *T. frauenfeldii*, and *B. sinensis* indicate succession within the retained larger-cell fraction toward diatoms favored under lower nutrient concentrations and stronger mixing (He et al., 2025).

Zooplankton showed a decoupling between numerical density and biomass from spring to summer. Similar densities but higher summer biomass indicate a shift toward larger-bodied taxa. The spring assemblage was dominated by *Calanus sinicus*, consistent with its preference for cool, nutrient-rich coastal waters and decline at temperatures above approximately 20 °C (Uye, 2000; Li et al., 2026). Summer dominance was distributed among multiple species, producing the highest zooplankton (*H*′). The autumn decline in zooplankton density likely reflected reduced food availability after the three-order-of-magnitude decrease in phytoplankton biomass.

Macrobenthos showed the strongest seasonal amplitude, with a 14-fold decline in density from summer to autumn and three azoic stations. Although bottom-layer DO had recovered by autumn, the benthic community may have retained a lagged signal of summer stress through mortality, impaired recruitment, delayed recolonization, substrate heterogeneity, or unmeasured disturbance history. The sharp reduction in species richness, from 138 species in summer to 33 in autumn, suggests that benthic recovery did not immediately track water-column reoxygenation, but the present design cannot isolate the responsible mechanism.

Diversity metrics therefore require compartment-specific interpretation. Phytoplankton (*H*′) increased when a single dominant species collapsed, despite reduced standing stock. In contrast, zooplankton and macrobenthic diversity declined with abundance, reflecting the loss of rare and specialist taxa. A single diversity index cannot serve as a universal indicator of ecosystem condition across compartments.

### 4.4 Environmental drivers across biological groups

CCA results identified DO and temperature as the only variables retained across all three biological groups, indicating that these parameters define common axes of seasonal ecosystem reorganization. The near-orthogonal DIN and DO vectors in the phytoplankton analysis suggest that nutrient enrichment and oxygen depletion explained complementary dimensions of community variation. Thus, the land–ocean nutrient gradient and the stratification-driven oxygen gradient appear to operate as partly independent forcing axes.

DIN and DIP were retained for both planktonic groups but not for macrobenthos. Phytoplankton respond directly to dissolved nutrients, while zooplankton respond indirectly through phytoplankton-mediated food quantity and quality. Macrobenthos integrate nutrient effects mainly through oxygen conditions and organic matter flux, both partly captured by DO and temperature. The non-selection of most sediment chemical variables should not be read as evidence that sediment processes were unimportant, but rather as a limitation of the measured predictor set and seasonal sampling design.

The lower explained variance for macrobenthos, 9.2%, compared with phytoplankton and zooplankton, indicates that the retained variables captured only a modest part of benthic variation. Unmeasured drivers, including larval supply, biological interactions, microhabitat heterogeneity, and legacy disturbance (Xu et al., 2017; Kim et al., 2019). SPM was not retained for any group, likely because most EEZ stations were seaward of the turbidity maximum, where SPM variation was insufficient to structure communities after accounting for temperature, nutrients, and oxygen.

### 4.5 Implications for ecological baselines and management

The integrated survey design provides a more robust ecological baseline than single-season or single-compartment assessments. In summer, elevated DIN, *S. costatum* near monodominance, bottom-water oxygen depletion, and sediment PHC and sulfide enrichment formed a coupled ecosystem state. Autumn represented a different configuration, characterized by widespread DIP exceedance, reduced biological standing stock, and lagged sedimentary TN and Hg enrichment. These contrasting states would not be captured by summer-focused monitoring alone.

Comparison with historical records suggests that 2024 DIN concentrations fall within the post-2003 plateau range (Liu et al., 2022), while DIP concentrations were slightly higher than declining trends reported for the estuary proper, possibly due to stronger benthic recycling at EEZ stations. The summer DO minimum of 2.10 mg L^−1^ is consistent with hypoxia documented since the early 2000s (Li et al., 2002; Wang et al., 2017). Sediment trace-metal concentrations were comparable to previous ECS shelf baselines (Cao et al., 2015; Xu et al., 2018; Wang et al., 2023). The dominance of *S. costatum* in spring–summer and *C. sinicus* in spring also agrees with long-term biological records (Wang L. et al., 2016; Xu et al., 2019; Sun et al., 2022; Li et al., 2026). The sharp autumn macrobenthic decline, however, has been less widely documented and warrants further study.

Several management implications can be drawn cautiously from these observations. Nutrient control should address both nitrogen and phosphorus, but with seasonal emphasis: nitrogen reduction is most relevant during the high-discharge period, whereas phosphorus management should consider the possibility of autumn benthic recycling. Continuous DO and temperature sensor arrays could provide a cost-effective monitoring backbone, supplemented by periodic ship-based surveys of nutrients, sediments, and biological communities. Because the net-collected phytoplankton assemblage responded strongly to seasonal nutrient and oxygen change, routine characterization of this microalgal component, and eventually its coupling with molecular or high-throughput microbial surveys, could provide an early indicator of ecosystem state. The autumn decline in macrobenthic standing stock suggests that the post-hypoxia recovery period may be a bottleneck for ecosystem resilience.

This study has limitations. Spring full-element biological sampling covered only 17 stations, and sediment data gaps prevented complete seasonal trajectories for Hg, TP, and TN. The CCA explained a modest fraction of macrobenthic variation, indicating that unmeasured drivers were important. The phytoplankton community was characterized from 77 μm net-collected material and morphological identification rather than from whole-water microscopy, flow cytometry, molecular, or high-throughput sequencing methods. Consequently, smaller phytoplankton, picophytoplankton, bacteria, archaea, and many non-pigmented protists were not resolved; coupling net-based morphological surveys with whole-water and molecular approaches in future work would refine the microbial-ecological interpretation. Finally, the study covered 1 year only; interannual variability in discharge, typhoon activity, and water-mass configuration may alter ecosystem states. Repeating this survey design over multiple years would help separate recurrent seasonal patterns from interannual variability and refine the ecological baseline for the Changjiang EEZ.

## Data Availability

The raw data supporting the conclusions of this article will be made available by the authors, without undue reservation.
