# Supplementary material for: Land–ocean nutrient gradients are associated with seasonal shifts in net-collected phytoplankton assemblages in the Yangtze River Estuary exclusive economic zone
Source: Front Microbiol. 2026 Aug 20;17:1897991. doi: 10.3389/fmicb.2026.1897991 (PMC13538444; doi:10.3389/fmicb.2026.1897991)
Supplement: Supplementary file 1 [file Table_1.DOCX]

**Supporting Information**

Land-ocean nutrient gradients are associated with seasonal shifts in net-collected phytoplankton assemblages in the Yangtze River Estuary exclusive economic zone

Bangping Deng^1^, Wei Liu^1,*^, Jun Li^1^, Tao Yang^1^, Jun Shi^1^, Yiming Yuan^1^, Shengming Cheng^2^, Lei Wu^3^, Chuxu Xiong^4^, Lichen Zhao^5^, Xiping Zheng^6^ and Lin Zhu^2^

^1^ East China Sea Ecology Center, and Key Laboratory of Marine Ecological Monitoring and Restoration Technologies, Ministry of Natural Resources, Shanghai 201206, China

^2^ China Three Gorges Investment Management Co., Ltd., Shanghai, China

^3^ Huaneng (Shanghai) Clean Energy Development Co., Ltd., Shanghai, China

^4^ Shanghai Electric Power Co., Ltd., Shanghai, China

^5^ Shanghai Electric Wind Power Group Co., Ltd., Shanghai, China

^6^ CRCC Harbour & Channel Engineering Bureau Group Co., Ltd., Guangdong, China

* Correspondence: Wei Liu, *memrt@ecs.mnr.gov.cn*

# Sampling metadata and coverage

**Table S1. Seasonal sampling coverage by data matrix.**

| **Season** | **Data matrix** | **Number of stations or station columns** | **Note** |
| --- | --- | --- | --- |
| Spring | Water quality | 28 | water-column records; multiple depths per station |
| Spring | Sediment | 17 | surficial sediment records |
| Spring | Phytoplankton | 17 | non-empty station columns in raw biological matrix |
| Spring | Zooplankton | 17 | non-empty station columns in raw biological matrix |
| Spring | Macrobenthos | 17 | non-empty station columns in raw biological matrix |
| Summer | Water quality | 28 | water-column records; multiple depths per station |
| Summer | Sediment | 28 | surficial sediment records |
| Summer | Phytoplankton | 28 | non-empty station columns in raw biological matrix |
| Summer | Zooplankton | 23 | non-empty station columns in raw biological matrix |
| Summer | Macrobenthos | 28 | non-empty station columns in raw biological matrix |
| Autumn | Water quality | 28 | water-column records; multiple depths per station |
| Autumn | Sediment | 28 | surficial sediment records |
| Autumn | Phytoplankton | 28 | non-empty station columns in raw biological matrix |
| Autumn | Zooplankton | 28 | non-empty station columns in raw biological matrix |
| Autumn | Macrobenthos | 25 | non-empty station columns in raw biological matrix |

Water-quality files contain multiple depth records per station. Biological counts are based on non-empty station columns in the available raw matrices.

**Table S2. Station metadata and biological sampling coverage.**

| **Station** | **Longitude E** | **Latitude N** | **Spring P/Z/M** | **Summer P/Z/M** | **Autumn P/Z/M** |
| --- | --- | --- | --- | --- | --- |
| S1 | 122.356 | 31.5 | -/-/- | Y/Y/Y | Y/Y/Y |
| S2 | 122.5 | 31.375 | Y/Y/Y | Y/Y/Y | Y/Y/Y |
| S3 | 122.5 | 31.5 | -/-/- | Y/-/Y | Y/Y/Y |
| S4 | 122.272 | 31.738 | Y/Y/Y | Y/Y/Y | Y/Y/Y |
| S5 | 122.523 | 31.743 | -/-/- | Y/Y/Y | Y/Y/- |
| S6 | 122.689 | 31.362 | -/-/- | Y/-/Y | Y/Y/Y |
| S7 | 122.683 | 31.23 | -/-/- | Y/Y/Y | Y/Y/Y |
| S8 | 122.689 | 31.606 | -/-/- | Y/-/Y | Y/Y/Y |
| S9 | 122.75 | 31.5 | Y/Y/Y | Y/Y/Y | Y/Y/Y |
| S10 | 122.75 | 31.75 | Y/Y/Y | Y/Y/Y | Y/Y/Y |
| S11 | 122.854 | 31.22 | Y/Y/Y | Y/Y/Y | Y/Y/Y |
| S12 | 122.856 | 31.605 | Y/Y/Y | Y/Y/Y | Y/Y/Y |
| S13 | 122.981 | 31.5 | -/-/- | Y/-/Y | Y/Y/Y |
| S14 | 122.983 | 31.742 | -/-/- | Y/Y/Y | Y/Y/Y |
| S15 | 123 | 31 | -/-/- | Y/-/Y | Y/Y/Y |
| S16 | 123.154 | 31.22 | -/-/- | Y/Y/Y | Y/Y/Y |
| S17 | 123.26 | 31.501 | Y/Y/Y | Y/Y/Y | Y/Y/Y |
| S18 | 123.26 | 31.743 | Y/Y/Y | Y/Y/Y | Y/Y/Y |
| S19 | 123.248 | 30.868 | Y/Y/Y | Y/Y/Y | Y/Y/Y |
| S20 | 123.451 | 31.221 | Y/Y/Y | Y/Y/Y | Y/Y/Y |
| S21 | 123.504 | 31.499 | Y/Y/Y | Y/Y/Y | Y/Y/Y |
| S22 | 123.503 | 31.743 | -/-/- | Y/Y/Y | Y/Y/Y |
| S23 | 123.501 | 30.868 | Y/Y/Y | Y/Y/Y | Y/Y/Y |
| S24 | 123.748 | 31.5 | Y/Y/Y | Y/Y/Y | Y/Y/- |
| S25 | 123.748 | 31.221 | Y/Y/Y | Y/Y/Y | Y/Y/Y |
| S26 | 123.75 | 31.743 | Y/Y/Y | Y/Y/Y | Y/Y/Y |
| S27 | 123.658 | 30.753 | Y/Y/Y | Y/Y/Y | Y/Y/- |
| S28 | 123.748 | 30.866 | Y/Y/Y | Y/Y/Y | Y/Y/Y |

P/Z/M = phytoplankton, zooplankton, and macrobenthos. Y indicates that the station is represented in the available biological raw matrix; - indicates that no non-empty station column was present in that matrix.

# Environmental and sediment summaries

**Table S3. Seasonal summary of water-quality variables.**

| **Parameter** | **Season** | **n** | **Mean** | **Median** | **SD** | **Min** | **Q1** | **Q3** | **Max** |
| --- | --- | --- | --- | --- | --- | --- | --- | --- | --- |
| pH | Spring | 85 | 8.287 | 8.26 | 0.173 | 8.07 | 8.13 | 8.41 | 8.7 |
| pH | Summer | 85 | 8.132 | 8.05 | 0.214 | 7.89 | 7.96 | 8.25 | 8.76 |
| pH | Autumn | 82 | 8.167 | 8.19 | 0.09 | 7.98 | 8.09 | 8.25 | 8.28 |
| DO | Spring | 85 | 9.509 | 9.4 | 1.765 | 7.02 | 8.01 | 10.4 | 14.4 |
| DO | Summer | 85 | 5.849 | 5.92 | 2.379 | 2.1 | 3.79 | 7.63 | 12.7 |
| DO | Autumn | 82 | 6.889 | 6.945 | 0.321 | 5.64 | 6.832 | 7.06 | 7.52 |
| COD_Mn_ | Spring | 85 | 1.026 | 0.93 | 0.477 | 0.38 | 0.65 | 1.25 | 2.57 |
| COD_Mn_ | Summer | 85 | 0.915 | 0.64 | 0.755 | 0.075 | 0.33 | 1.53 | 4.22 |
| COD_Mn_ | Autumn | 82 | 0.481 | 0.42 | 0.227 | 0.18 | 0.352 | 0.58 | 1.77 |
| DIN | Spring | 73 | 0.232 | 0.193 | 0.154 | 0.014 | 0.122 | 0.284 | 0.702 |
| DIN | Summer | 85 | 0.283 | 0.205 | 0.298 | 0.041 | 0.09 | 0.284 | 1.361 |
| DIN | Autumn | 82 | 0.143 | 0.148 | 0.04 | 0.064 | 0.119 | 0.172 | 0.24 |
| DIP | Spring | 85 | 0.011 | 0.007 | 0.006 | 0.004 | 0.005 | 0.016 | 0.024 |
| DIP | Summer | 85 | 0.017 | 0.016 | 0.008 | 0.004 | 0.009 | 0.024 | 0.035 |
| DIP | Autumn | 82 | 0.021 | 0.021 | 0.004 | 0.013 | 0.018 | 0.023 | 0.035 |
| SPM | Spring | 85 | 14.551 | 8.5 | 18.322 | 2.9 | 5.5 | 15.3 | 127 |
| SPM | Summer | 85 | 9.821 | 8 | 7.244 | 2.1 | 5 | 13 | 42.7 |
| SPM | Autumn | 82 | 31.654 | 18.2 | 72.261 | 10.4 | 14.675 | 22.3 | 560 |

Values are reproduced from 03-res/water_quality_summary_by_season.csv.

**Table S4. Seasonal summary of sediment variables.**

| **Parameter** | **Season** | **n** | **Mean** | **Median** | **SD** | **Min** | **Q1** | **Q3** | **Max** |
| --- | --- | --- | --- | --- | --- | --- | --- | --- | --- |
| OC | Spring | 17 | 0.227 | 0.19 | 0.131 | 0.1 | 0.13 | 0.3 | 0.61 |
| OC | Summer | 28 | 0.344 | 0.355 | 0.107 | 0.14 | 0.278 | 0.395 | 0.58 |
| OC | Autumn | 28 | 0.351 | 0.32 | 0.159 | 0.13 | 0.228 | 0.492 | 0.67 |
| PHC | Spring | 11 | 22.34 | 9.52 | 25.343 | 3.26 | 5.39 | 30.8 | 88.3 |
| PHC | Summer | 28 | 76.948 | 80.25 | 44.354 | 5.9 | 45.8 | 114.25 | 180 |
| PHC | Autumn | 23 | 43.913 | 24.5 | 44.895 | 3.89 | 11.45 | 52.85 | 150 |
| Sulfide | Spring | 17 | 22.776 | 22.4 | 4.345 | 13.6 | 20.5 | 24.3 | 34.2 |
| Sulfide | Summer | 28 | 44.789 | 44.95 | 13.819 | 19.8 | 37.425 | 49.05 | 80.8 |
| Sulfide | Autumn | 28 | 32.55 | 32.25 | 7.044 | 19.4 | 28.275 | 36.425 | 52.4 |
| Cu | Spring | 17 | 7.704 | 5.74 | 5.845 | 3.45 | 4.06 | 8.01 | 24.8 |
| Cu | Summer | 28 | 12.216 | 12.6 | 5.319 | 3.74 | 7.817 | 15.425 | 26 |
| Cu | Autumn | 28 | 14.21 | 12.4 | 7.756 | 4.64 | 8.085 | 18.65 | 30.9 |
| Zn | Spring | 17 | 38.994 | 36.5 | 8.198 | 29.8 | 34.8 | 40 | 63.2 |
| Zn | Summer | 28 | 51.8 | 51.55 | 12.472 | 36.8 | 39.875 | 57.925 | 85.6 |
| Zn | Autumn | 28 | 48.296 | 45.05 | 15.261 | 24.4 | 37.35 | 52.9 | 84 |
| Hg | Spring | 16 | 0.008 | 0.006 | 0.006 | 0.003 | 0.004 | 0.011 | 0.027 |
| Hg | Summer | 0 |  |  |  |  |  |  |  |
| Hg | Autumn | 27 | 0.024 | 0.022 | 0.011 | 0.007 | 0.016 | 0.032 | 0.055 |
| TP | Spring | 0 |  |  |  |  |  |  |  |
| TP | Summer | 28 | 522.821 | 481.5 | 150.06 | 276 | 450.5 | 584.25 | 1030 |
| TP | Autumn | 28 | 367.171 | 359.168 | 110.15 | 212.004 | 281.228 | 440.147 | 601.06 |
| TN | Spring | 0 |  |  |  |  |  |  |  |
| TN | Summer | 28 | 74.611 | 64.15 | 26.475 | 35.5 | 57.875 | 95.75 | 138 |
| TN | Autumn | 28 | 115.586 | 126.068 | 37.329 | 49.733 | 80.531 | 142.149 | 169.522 |

Values are reproduced from 03-res/sediment_descriptive_stats.csv.

# Biological community summaries

**Table S5. Seasonal summaries of station-level biological metrics.**

| **Group** | **Season** | **Metric** | **n** | **Mean** | **Median** | **Min** | **Max** |
| --- | --- | --- | --- | --- | --- | --- | --- |
| Phytoplankton | Spring | total_density | 17 | 73160507 | 11162897 | 271726 | 474155260 |
| Phytoplankton | Spring | dominant_contribution | 17 | 63.979 | 65.447 | 30.767 | 90.416 |
| Phytoplankton | Spring | shannon | 17 | 1.104 | 1.077 | 0.459 | 1.916 |
| Phytoplankton | Spring | taxa_richness | 17 | 26.059 | 26 | 19 | 39 |
| Phytoplankton | Summer | total_density | 28 | 53379672 | 897700 | 14255 | 554520806 |
| Phytoplankton | Summer | dominant_contribution | 28 | 72.008 | 72.919 | 27.471 | 99.926 |
| Phytoplankton | Summer | shannon | 28 | 0.819 | 0.874 | 0.007 | 2.089 |
| Phytoplankton | Summer | taxa_richness | 28 | 22.643 | 23 | 9 | 50 |
| Phytoplankton | Autumn | total_density | 28 | 40010 | 22992 | 1193 | 237542 |
| Phytoplankton | Autumn | dominant_contribution | 28 | 41.217 | 39.795 | 16.667 | 90.079 |
| Phytoplankton | Autumn | shannon | 28 | 1.873 | 1.929 | 0.504 | 2.631 |
| Phytoplankton | Autumn | taxa_richness | 28 | 20.929 | 20.5 | 8 | 37 |
| Zooplankton | Spring | zoo_density | 17 | 465.195 | 537.052 | 77.929 | 1159 |
| Zooplankton | Spring | shannon | 17 | 0.919 | 0.923 | 0.461 | 1.748 |
| Zooplankton | Spring | taxa_richness | 17 | 16.471 | 16 | 13 | 20 |
| Zooplankton | Spring | zoo_biomass | 17 | 293.935 | 303.4 | 85.1 | 513.6 |
| Zooplankton | Spring | density_from_biomass_table | 17 | 465.196 | 537.05 | 77.93 | 1159 |
| Zooplankton | Spring | richness_from_biomass_table | 17 | 16.471 | 16 | 13 | 20 |
| Zooplankton | Summer | zoo_density | 23 | 414.448 | 381.12 | 104.536 | 1054 |
| Zooplankton | Summer | shannon | 23 | 2.292 | 2.425 | 0.63 | 2.836 |
| Zooplankton | Summer | taxa_richness | 23 | 32.913 | 35 | 12 | 50 |
| Zooplankton | Summer | zoo_biomass | 23 | 642.088 | 550.68 | 39.45 | 1348 |
| Zooplankton | Summer | density_from_biomass_table | 23 | 414.448 | 381.12 | 104.54 | 1054 |
| Zooplankton | Summer | richness_from_biomass_table | 23 | 32.913 | 35 | 12 | 50 |
| Zooplankton | Autumn | zoo_density | 28 | 40.444 | 32.767 | 9.787 | 330.715 |
| Zooplankton | Autumn | shannon | 28 | 1.956 | 2.079 | 0.192 | 2.5 |
| Zooplankton | Autumn | taxa_richness | 28 | 13.5 | 13 | 6 | 20 |
| Zooplankton | Autumn | zoo_biomass | 28 | 205.389 | 82.85 | 18.6 | 2907 |
| Zooplankton | Autumn | density_from_biomass_table | 28 | 40.445 | 32.77 | 9.79 | 330.72 |
| Zooplankton | Autumn | richness_from_biomass_table | 28 | 13.5 | 13 | 6 | 20 |
| Macrobenthos | Spring | macrobenthos_density | 17 | 249.39 | 193.31 | 13.32 | 966.66 |
| Macrobenthos | Spring | taxa_richness | 17 | 18.588 | 17 | 4 | 37 |
| Macrobenthos | Spring | shannon | 17 | 2.182 | 2.21 | 1.134 | 3.134 |
| Macrobenthos | Spring | macrobenthos_biomass | 17 | 6.234 | 4.43 | 0.267 | 32.678 |
| Macrobenthos | Summer | macrobenthos_density | 28 | 533.066 | 324.98 | 30 | 1830 |
| Macrobenthos | Summer | taxa_richness | 28 | 29.571 | 27 | 5 | 73 |
| Macrobenthos | Summer | shannon | 28 | 2.562 | 2.903 | 0.758 | 3.845 |
| Macrobenthos | Summer | macrobenthos_biomass | 28 | 25.551 | 16.777 | 0.901 | 262.611 |
| Macrobenthos | Autumn | macrobenthos_density | 28 | 37.136 | 19.995 | 0 | 263.31 |
| Macrobenthos | Autumn | taxa_richness | 28 | 4.179 | 3.5 | 0 | 18 |
| Macrobenthos | Autumn | shannon | 25 | 1.068 | 1.099 | 0 | 2.572 |
| Macrobenthos | Autumn | macrobenthos_biomass | 28 | 1.809 | 0.198 | 0 | 14.525 |

**Table S6. Top ten taxa by total abundance or density within each biological matrix.**

| **Group** | **Season** | **Rank** | **Accepted Latin name** | **Total** | **Contribution %** |
| --- | --- | --- | --- | --- | --- |
| Phytoplankton | Spring | 1 | *Skeletonema costatum* | 948683000 | 76.28 |
| Phytoplankton | Spring | 2 | *Chaetoceros curvisetus* | 168793960 | 13.57 |
| Phytoplankton | Spring | 3 | *Pseudo-nitzschia pungens* | 57534400 | 4.63 |
| Phytoplankton | Spring | 4 | *Chaetoceros castracanei* | 17869990 | 1.44 |
| Phytoplankton | Spring | 5 | *Asterionella japonica* | 17113321 | 1.38 |
| Phytoplankton | Spring | 6 | *Chaetoceros debilis* | 14502280 | 1.17 |
| Phytoplankton | Spring | 7 | *Rhizosolenia setigera* | 6290010 | 0.51 |
| Phytoplankton | Spring | 8 | *Chaetoceros affinis var. affinis* | 5595000 | 0.45 |
| Phytoplankton | Spring | 9 | *Chaetoceros lorenzianus* | 3242249 | 0.26 |
| Phytoplankton | Spring | 10 | *Chaetoceros pseudocurvisetus* | 838510 | 0.07 |
| Phytoplankton | Summer | 1 | *Skeletonema costatum* | 1470145980 | 98.36 |
| Phytoplankton | Summer | 2 | *Pseudo-nitzschia pungens* | 16787181 | 1.12 |
| Phytoplankton | Summer | 3 | *Noctiluca scintillans* | 2605614 | 0.17 |
| Phytoplankton | Summer | 4 | *Chaetoceros lorenzianus* | 2137881 | 0.14 |
| Phytoplankton | Summer | 5 | *Pseudo-nitzschia delicatissima* | 852287 | 0.06 |
| Phytoplankton | Summer | 6 | *Chaetoceros debilis* | 527216 | 0.04 |
| Phytoplankton | Summer | 7 | *Thalassiosira binata* | 297990 | 0.02 |
| Phytoplankton | Summer | 8 | *Chaetoceros comperssus* | 194700 | 0.01 |
| Phytoplankton | Summer | 9 | *Thalassionema nitzschioides* | 128820 | 0.01 |
| Phytoplankton | Summer | 10 | *Ceratium fusus* | 61373 | 0 |
| Phytoplankton | Autumn | 1 | *Coscinodiscus wailesii* | 340422 | 30.39 |
| Phytoplankton | Autumn | 2 | *Thalassiothrix frauenfeldii* | 274712 | 24.52 |
| Phytoplankton | Autumn | 3 | *Biddulphia sinensis* | 194886 | 17.4 |
| Phytoplankton | Autumn | 4 | *Thalassionema nitzschioides* | 53161 | 4.75 |
| Phytoplankton | Autumn | 5 | *Coscinodiscus jonesianus* | 46166 | 4.12 |
| Phytoplankton | Autumn | 6 | *Coscinodiscus subtilis* | 32277 | 2.88 |
| Phytoplankton | Autumn | 7 | *Chaetoceros lorenzianus* | 19874 | 1.77 |
| Phytoplankton | Autumn | 8 | *Triceratium favus* | 18102 | 1.62 |
| Phytoplankton | Autumn | 9 | *Coscinodiscus asteromphalus* | 16971 | 1.51 |
| Phytoplankton | Autumn | 10 | *Tortula rivularis* | 14640 | 1.31 |
| Zooplankton | Spring | 1 | *Calanus sinicus Brodsky* | 6293 | 79.58 |
| Zooplankton | Spring | 2 | *Euphausiacea larva* | 885.425 | 11.2 |
| Zooplankton | Spring | 3 | *Medusa larva* | 165.026 | 2.09 |
| Zooplankton | Spring | 4 | *Sagitta nagae Alvarino* | 76.552 | 0.97 |
| Zooplankton | Spring | 5 | *Euchaeta plana Mori* | 65.936 | 0.83 |
| Zooplankton | Spring | 6 | *Mysidacea larva* | 50.446 | 0.64 |
| Zooplankton | Spring | 7 | *Chaetognatha larva* | 46.493 | 0.59 |
| Zooplankton | Spring | 8 | *Oikopleura dioica Fol* | 44.378 | 0.56 |
| Zooplankton | Spring | 9 | *Labidocera euchaeta Giesbrecht* | 39.557 | 0.5 |
| Zooplankton | Spring | 10 | *Muggiaea atlantica Cunningham* | 37.079 | 0.47 |
| Zooplankton | Summer | 1 | *Calanus sinicus Brodsky* | 1709 | 17.93 |
| Zooplankton | Summer | 2 | *Brachyura zoea larva* | 1126 | 11.81 |
| Zooplankton | Summer | 3 | *Sagitta enflata Grassi* | 1095 | 11.49 |
| Zooplankton | Summer | 4 | *Diphyes chamissonis Huxley* | 985.742 | 10.34 |
| Zooplankton | Summer | 5 | *Lucifer intermedius Hansen* | 487.527 | 5.11 |
| Zooplankton | Summer | 6 | *Eucalanus subcrassus Giesbrecht* | 443.51 | 4.65 |
| Zooplankton | Summer | 7 | *Doliolum gegenbauri Uljanin* | 390.81 | 4.1 |
| Zooplankton | Summer | 8 | *Penilia avirostris Dana* | 387.55 | 4.07 |
| Zooplankton | Summer | 9 | *Muggiaea atlantica Cunningham* | 309.28 | 3.24 |
| Zooplankton | Summer | 10 | *Macrura larva* | 304.107 | 3.19 |
| Zooplankton | Autumn | 1 | *Euphausiacea larva* | 465.246 | 41.08 |
| Zooplankton | Autumn | 2 | *Euchaeta concinna Dana* | 106.344 | 9.39 |
| Zooplankton | Autumn | 3 | *Calanus sinicus Brodsky* | 93.5 | 8.26 |
| Zooplankton | Autumn | 4 | *Diphyes chamissonis Huxley* | 90.716 | 8.01 |
| Zooplankton | Autumn | 5 | *Sagitta enflata Grassi* | 78.737 | 6.95 |
| Zooplankton | Autumn | 6 | *Chaetognatha larva* | 74.243 | 6.56 |
| Zooplankton | Autumn | 7 | *Cypridina acuminata Muller* | 53.611 | 4.73 |
| Zooplankton | Autumn | 8 | *Subeucalanus subcrassus Giesbrecht* | 50.034 | 4.42 |
| Zooplankton | Autumn | 9 | *Mysidacea larva* | 32.46 | 2.87 |
| Zooplankton | Autumn | 10 | *Medusa larva* | 25.441 | 2.25 |
| Macrobenthos | Spring | 1 | *Heteromastus filiformis* | 706.67 | 16.67 |
| Macrobenthos | Spring | 2 | *Prionospio queenslandica* | 419.98 | 9.91 |
| Macrobenthos | Spring | 3 | *Moerella jedoensis* | 170 | 4.01 |
| Macrobenthos | Spring | 4 | *Ophelina acuminata* | 153.31 | 3.62 |
| Macrobenthos | Spring | 5 | *Aricidea fragilis* | 143.35 | 3.38 |
| Macrobenthos | Spring | 6 | *Nucula japonica* | 140 | 3.3 |
| Macrobenthos | Spring | 7 | *Nucula faba* | 133.34 | 3.15 |
| Macrobenthos | Spring | 8 | *Paralacydonia paradoxa* | 123.32 | 2.91 |
| Macrobenthos | Spring | 9 | *Lumbrineris latreilli* | 119.99 | 2.83 |
| Macrobenthos | Spring | 10 | *Nuttallia minuta* | 119.98 | 2.83 |
| Macrobenthos | Summer | 1 | *Sabellaria asiatica* | 1873 | 12.55 |
| Macrobenthos | Summer | 2 | *Prionospio queenslandica* | 980 | 6.57 |
| Macrobenthos | Summer | 3 | *Sthenolepis japonica* | 599.99 | 4.02 |
| Macrobenthos | Summer | 4 | *Xenophthalmus pinnotheroides* | 593.32 | 3.98 |
| Macrobenthos | Summer | 5 | *Heteromastus filiformis* | 463.33 | 3.1 |
| Macrobenthos | Summer | 6 | *Nuttallia minuta* | 433.33 | 2.9 |
| Macrobenthos | Summer | 7 | *Lumbrineris cruzensis* | 356.67 | 2.39 |
| Macrobenthos | Summer | 8 | *Cirratulus filiformis* | 346.64 | 2.32 |
| Macrobenthos | Summer | 9 | *Nassarius variciferus* | 316.67 | 2.12 |
| Macrobenthos | Summer | 10 | *Apionsoma trichocephala* | 313.32 | 2.1 |
| Macrobenthos | Autumn | 1 | *Heteromastus filiformis* | 286.67 | 27.57 |
| Macrobenthos | Autumn | 2 | *Prionospio queenslandica* | 259.98 | 25 |
| Macrobenthos | Autumn | 3 | *Apionsoma trichocephala* | 60 | 5.77 |
| Macrobenthos | Autumn | 4 | *Lumbrineris cruzensis* | 53.33 | 5.13 |
| Macrobenthos | Autumn | 5 | *Xenophthalmus pinnotheroides* | 50 | 4.81 |
| Macrobenthos | Autumn | 6 | *Amphiura vadicola* | 40 | 3.85 |
| Macrobenthos | Autumn | 7 | *Glycera chirori* | 39.99 | 3.85 |
| Macrobenthos | Autumn | 8 | *Chaetozone setosa* | 19.99 | 1.92 |
| Macrobenthos | Autumn | 9 | *Lumbrineris heteropoda* | 16.66 | 1.6 |
| Macrobenthos | Autumn | 10 | *Glycinde gurjanovae* | 16.65 | 1.6 |

Contributions are percentages of the summed total within each group-season matrix.

# Seasonal statistical tests

**Table S7. Kruskal-Wallis tests for water-quality and sediment variables.**

| **Dataset** | **Parameter** | **H** | **df** | **p** | **Significant** |
| --- | --- | --- | --- | --- | --- |
| Water quality | pH | 38.146 | 2 | 5.21e-09 | Yes |
| Water quality | DO | 131.294 | 2 | 3.09e-29 | Yes |
| Water quality | COD_Mn_ | 61.169 | 2 | 5.21e-14 | Yes |
| Water quality | DIN | 18.643 | 2 | 8.95e-05 | Yes |
| Water quality | DIP | 75.811 | 2 | 3.45e-17 | Yes |
| Water quality | SPM | 75.671 | 2 | 3.70e-17 | Yes |
| Sediment | OC | 11.83 | 2 | 0.0027 | Yes |
| Sediment | PHC | 14.008 | 2 | 9.08e-04 | Yes |
| Sediment | Sulfide | 36.8 | 2 | 1.02e-08 | Yes |
| Sediment | Cu | 13.666 | 2 | 0.0011 | Yes |
| Sediment | Zn | 14.378 | 2 | 7.55e-04 | Yes |
| Sediment | Hg | 23.518 | 1 | 1.24e-06 | Yes |
| Sediment | TP | 16.383 | 1 | 5.17e-05 | Yes |
| Sediment | TN | 15.597 | 1 | 7.84e-05 | Yes |

**Table S8. Kruskal-Wallis tests for biological station-level metrics.**

| **Group** | **Metric** | **n** | **Statistic** | **df** | **p** | **Method** |
| --- | --- | --- | --- | --- | --- | --- |
| Phytoplankton | density | 73 | 49.424 | 2 | 1.85e-11 | Kruskal-Wallis |
| Phytoplankton | dominant_contribution | 73 | 26.818 | 2 | 1.50e-06 | Kruskal-Wallis |
| Phytoplankton | shannon | 73 | 31.556 | 2 | 1.41e-07 | Kruskal-Wallis |
| Zooplankton | density | 68 | 46.423 | 2 | 8.30e-11 | Kruskal-Wallis |
| Zooplankton | biomass | 68 | 36.11 | 2 | 1.44e-08 | Kruskal-Wallis |
| Zooplankton | shannon | 68 | 37.796 | 2 | 6.20e-09 | Kruskal-Wallis |
| Macrobenthos | density | 73 | 41.813 | 2 | 8.32e-10 | Kruskal-Wallis |
| Macrobenthos | biomass | 73 | 36.193 | 2 | 1.38e-08 | Kruskal-Wallis |
| Macrobenthos | shannon | 70 | 30.979 | 2 | 1.88e-07 | Kruskal-Wallis |

**Table S9. Available pairwise post hoc outputs.**

| **Dataset or group** | **Parameter or metric** | **Comparison** | **Statistic** | **Adjusted p** | **Significance** |
| --- | --- | --- | --- | --- | --- |
| Water quality | pH | Autumn - Spring | -3.819 | 4.02e-04 | Dunn |
| Water quality | pH | Autumn - Summer | 2.234 | 0.0764 | Dunn |
| Water quality | pH | Spring - Summer | 6.109 | 3.02e-09 | Dunn |
| Water quality | DO | Autumn - Spring | -9.369 | 2.19e-20 | Dunn |
| Water quality | DO | Autumn - Summer | 0.909 | 1 | Dunn |
| Water quality | DO | Spring - Summer | 10.372 | 9.97e-25 | Dunn |
| Water quality | COD_Mn_ | Autumn - Spring | -7.819 | 1.60e-14 | Dunn |
| Water quality | COD_Mn_ | Autumn - Summer | -4.145 | 1.02e-04 | Dunn |
| Water quality | COD_Mn_ | Spring - Summer | 3.707 | 6.28e-04 | Dunn |
| Water quality | DIN | Autumn - Spring | -3.799 | 4.37e-04 | Dunn |
| Water quality | DIN | Autumn - Summer | -3.65 | 7.87e-04 | Dunn |
| Water quality | DIN | Spring - Summer | 0.29 | 1 | Dunn |
| Water quality | DIP | Autumn - Spring | 8.612 | 2.16e-17 | Dunn |
| Water quality | DIP | Autumn - Summer | 3.279 | 0.0031 | Dunn |
| Water quality | DIP | Spring - Summer | -5.382 | 2.21e-07 | Dunn |
| Water quality | SPM | Autumn - Spring | 6.716 | 5.61e-11 | Dunn |
| Water quality | SPM | Autumn - Summer | 8.177 | 8.71e-16 | Dunn |
| Water quality | SPM | Spring - Summer | 1.475 | 0.4209 | Dunn |
| Sediment | OC | Autumn - Spring | 2.87 | 0.0123 | Dunn |
| Sediment | OC | Autumn - Summer | -0.46 | 1 | Dunn |
| Sediment | OC | Spring - Summer | -3.27 | 0.0032 | Dunn |
| Sediment | PHC | Autumn - Spring | 1.481 | 0.4159 | Dunn |
| Sediment | PHC | Autumn - Summer | -2.493 | 0.038 | Dunn |
| Sediment | PHC | Spring - Summer | -3.497 | 0.0014 | Dunn |
| Sediment | Sulfide | Autumn - Spring | 3.285 | 0.0031 | Dunn |
| Sediment | Sulfide | Autumn - Summer | -3.165 | 0.0047 | Dunn |
| Sediment | Sulfide | Spring - Summer | -6.036 | 4.73e-09 | Dunn |
| Sediment | Cu | Autumn - Spring | 3.52 | 0.0013 | Dunn |
| Sediment | Cu | Autumn - Summer | 0.513 | 1 | Dunn |
| Sediment | Cu | Spring - Summer | -3.074 | 0.0063 | Dunn |
| Sediment | Zn | Autumn - Spring | 2.464 | 0.0412 | Dunn |
| Sediment | Zn | Autumn - Summer | -1.524 | 0.3823 | Dunn |
| Sediment | Zn | Spring - Summer | -3.789 | 4.53e-04 | Dunn |
| Sediment | Hg | Autumn - Spring | 4.849 | 1.24e-06 | Dunn |
| Sediment | TP | Autumn - Summer | -4.048 | 5.17e-05 | Dunn |
| Sediment | TN | Autumn - Summer | 3.949 | 7.84e-05 | Dunn |
| Phytoplankton | density | Spring - Summer | 348 | 0.009 | ** |
| Phytoplankton | density | Spring - Autumn | 476 | 5.43e-12 | **** |
| Phytoplankton | density | Summer - Autumn | 754 | 1.12e-11 | **** |
| Phytoplankton | dominant_contribution | Spring - Summer | 190 | 0.269 | ns |
| Phytoplankton | dominant_contribution | Spring - Autumn | 396 | 1.66e-04 | *** |
| Phytoplankton | dominant_contribution | Summer - Autumn | 684 | 9.18e-07 | **** |
| Phytoplankton | shannon | Spring - Summer | 315 | 0.073 | ns |
| Phytoplankton | shannon | Spring - Autumn | 60 | 1.30e-05 | **** |
| Phytoplankton | shannon | Summer - Autumn | 86 | 1.76e-07 | **** |
| Zooplankton | density | Spring - Summer | 218 | 0.551 | ns |
| Zooplankton | density | Spring - Autumn | 470 | 8.16e-11 | **** |
| Zooplankton | density | Summer - Autumn | 636 | 2.04e-12 | **** |
| Zooplankton | biomass | Spring - Summer | 61 | 1.15e-04 | *** |
| Zooplankton | biomass | Spring - Autumn | 414 | 1.72e-05 | **** |
| Zooplankton | biomass | Summer - Autumn | 589 | 8.40e-08 | **** |
| Zooplankton | shannon | Spring - Summer | 17 | 4.10e-08 | **** |
| Zooplankton | shannon | Spring - Autumn | 24 | 3.96e-08 | **** |
| Zooplankton | shannon | Summer - Autumn | 505 | 3.67e-04 | *** |
| Macrobenthos | density | Spring - Summer | 152 | 0.045 | * |
| Macrobenthos | density | Spring - Autumn | 430.5 | 1.03e-05 | **** |
| Macrobenthos | density | Summer - Autumn | 754 | 9.36e-09 | **** |
| Macrobenthos | biomass | Spring - Summer | 122 | 0.006 | ** |
| Macrobenthos | biomass | Spring - Autumn | 397 | 3.09e-04 | *** |
| Macrobenthos | biomass | Summer - Autumn | 730 | 9.57e-08 | **** |
| Macrobenthos | shannon | Spring - Summer | 159.5 | 0.068 | ns |
| Macrobenthos | shannon | Spring - Autumn | 370 | 8.52e-05 | **** |
| Macrobenthos | shannon | Summer - Autumn | 633.5 | 1.37e-06 | **** |
